# Simulating water and salt changes in the root zone of salt–alkali fragrant pear and the selection of the optimal surface drip irrigation mode

**DOI:** 10.3389/fpls.2024.1455188

**Published:** 2024-12-11

**Authors:** Yanjie Li, Ping Gong, Xinlin He, Hongguang Liu, Zhijie Li, Ling Li, Chunxia Wang, Qiang Xu, Quan Chen, Jie Wei, Ping Lin, Xuyong Yu

**Affiliations:** ^1^ College of Water Conservancy and Architectural Engineering, Shihezi University, Shihezi, China; ^2^ Key Laboratory of Modern Water-Saving Irrigation of Xinjiang Production and Construction Group, Shihezi, China; ^3^ Hydrology and Water Resources Management Center of the Second Division of Xinjiang Production and Construction Corps, Tiemenguan, Xinjiang, China; ^4^ Agricultural Science Research Institute of the Second Division of the Xinjiang Production and Construction Corps, Tiemenguan, Xinjiang, China; ^5^ Xinjiang Tianye Co., Ltd., Shihezi, Xinjiang, China; ^6^ Hydrology and Water Resources Management Centre of the Eighth Division of Shihezi City, Shihezi, China

**Keywords:** HYDRUS-2D, root zone environment, water-salt changes, multi-objective optimization, Sailt-Alkali fruit trees

## Abstract

Faced with the increasingly serious problem of water scarcity, developing precise irrigation strategies for crops in saline alkali land can effectively reduce the negative effects of low water resource utilization. Using a model to simulate the dynamic changes in soil water and salt environment in the root zone of fragrant pear trees in saline alkali land, and verifying them from a production practice perspective with comprehensive benefits as the goal, can optimize the irrigation amount and irrigation technology elements of saline alkali fruit trees, broaden the comprehensive evaluation perspective of decision-makers, and have important significance for improving the yield and production efficiency of forestry and fruit industry in arid and semi-arid areas worldwide. In this study, a two-year field experiment based on three irrigation levels (3000, 3750, and 4500 m^3^·ha^−1^) and four emitter discharge rates (1, 2, 3, and 4 L·h^−1^) was conducted in Xinjiang, China. The root zone soil water content (SWC) and soil salinity content (SSC) dynamics were simulated during the fertility period of fragrant pear using the numerical model HYDRUS-2D and field data. The results showed that the R^2^, root mean squared error (RMSE), and Nash–Sutcliffe efficiency coefficient (NSE) of the HYDRUS-2D simulated soil water content (SWC) (soil salinity content SSC) reached 0.89–0.97 (0.91–0.97), 0.02–0.16 cm^3^·cm^-3^ (0.22–1.54 g·kg^−1^), and 0.76–0.95 (0.68–0.96), respectively, indicating the strong performance of the model. A positive correlation was observed between the irrigation amount and soil infiltration depth. Moderately increasing irrigation amount could effectively leach soil salinity at a depth of 80–100 cm and maintain a water and salt environment in the main root zone of 0–80 cm, benefiting the growth and development of the main root system of fragrant pear, as well as the yield and quality of above-ground fruits. The irrigation amount and emitter discharge were optimized and quantified based on multi-objective optimization methods, normalization processing, and spatial analysis methods to maximize yield, fruit weight, soluble solids, and net profits. When the yield, fruit weight, soluble solids, and net profits simultaneously reached 90% of their maximum value, the irrigation amount and emitter discharge ranges were 4274–4297 m^3^·ha^−1^ and 3.79–3.88 L·h^−1^, respectively. Our study provides new insights into regulating soil water and salt environmental factors in the saline fragrant pear root zone and assessing the impact of soil water and salt management under precision irrigation strategies, and profoundly influences decision-making for irrigation of forest fruits in saline arid zones based on a production practice perspective.

## 1 Introduction

The efficient utilization of water resources is a key element in maintaining sustainable agricultural development, which directly affects agricultural production, ecological environment, and socio-economic development (Zhang et al., 2023). However, for the southern Xinjiang region, which has long been constrained by drought and salinity, the equilibrium relationship between the water–salt environment of crop inter-root soils and the efficient utilization of water resources is more complex (Wang et al., 2024). A suitable inter-root water and salinity environment is crucial for crop yield in agricultural production. In addition, inter-root soil water and salinity levels regulate root growth and development, which affect nutrient uptake and above-ground crop fruit yield and quality (Zhang et al., 2022a; Zhang et al., 2022b). Therefore, optimizing irrigation technology parameters is important to balance the trade-off between the efficient use of water resources and the salt-leaching effect.

Irrigation is an important external driving factor that affects the water and salt environment in the root zone of saline alkali fruit trees. By influencing the root zone environment, the water and salt stress on the root zone can be alleviated. The distribution of soil water and salt is significantly affected by irrigation amount and irrigation technology parameters. Optimizing irrigation technology parameters can effectively reduce root zone salinity and enhance the competitiveness and stress resistance of fruit tree root resources (Gao et al., 2023; Zhang et al., 2022b). Irrigation quota, emitter discharge, emitter spacing, soil texture, etc. can all have an impact on the soil environment in the root zone of fruit trees (Brighenti et al., 2024; Yang et al., 2013). Emitter discharge and irrigation amount are key factors affecting soil water and salt distribution. On the one hand, increasing irrigation amount and emitter discharge is beneficial for improving vertical water infiltration and expanding low salt distribution areas (Tan et al., 2022), and promoting salt leaching to reduce pH and alleviate salt stress on roots. Scholars have demonstrated that the spatial distribution of wetted areas formed by different emitter discharges is complex, with an increase in emitter discharge enhancing the horizontal wetted area and reducing the vertical distribution range. More specifically, a large emitter discharge tends to form a ‘wide and shallow’ wetted area, while a small emitter discharge forms a ‘narrow and deep’ wetted area (Li and Kang, 2006). On the other hand, the distribution of root systems varies among different crops, and the range of moisture can significantly affect the morphology and water absorption activity of plant roots (Lin et al., 2018). In recent years, the application effect of drip irrigation on fruit tree yield and quality has received widespread attention. A meta-analysis quantitatively explained that moderately increasing irrigation amount can improve yield by 6.71% and water use efficiency by 119.0% (Cheng et al., 2023). However, there is little research on the interaction between irrigation amount and drip head flow rate on fruit trees in saline alkali arid areas. Therefore, exploring the adaptability of the two to the water and salt environment in the root zone of fruit trees is the key to optimizing drip irrigation technology parameters.

Considering the complexity of external factors, numerical simulation provides an effective and convenient solution to quantify the transport and distribution of soil moisture and salinity under different complex conditions. Among numerous water and salt transport models, HYDRUS (2D/3D) has been widely used in the dynamic simulation of soil moisture and salinity due to its flexible boundary condition settings and accurate simulation results (Erazo-Mesa et al., 2022; Liu Y et al., 2021; Liu H. et al., 2021). Liu Y. et al. (2021) performed the numerical simulation of soil water and salt changes in Xinjiang farmland under membrane drip irrigation and subsurface pipe drainage using the HYDRUS-2D model. The authors found that the HYDRUS-2D model performed better in predicting the water and salt transport trends of farmland soil profiles in the arid zone and the process of soil salt dynamics during the reproductive period of crops. Nazari et al. (2021) revealed the simulation of soil moisture changes and root water uptake in subsurface drip irrigation of apple trees to be in good agreement with the observational results. Given that different irrigation quotas and emitter discharge conditions exert different effects on the water–salt environment in the root zone of saline fruit trees, it is crucial to comprehensively explore the influence of irrigation technology parameters on the distribution of water and salt in the root zone.

Developing precise irrigation strategies not only requires exploring changes in the water and salt environment of the root zone, but also evaluating irrigation effectiveness through the response of aboveground growth, reflecting the most direct indicators of plant physiological changes, namely yield and fruit quality (Li L. et al., 2023). Previous studies have shown that irrigation can balance water relationships by altering plant physiology and structure, including tissue water content, water potential, gas exchange, and cell expansion. On the one hand, it can effectively inhibit leaf electrolyte leakage, improve plant physiological activity and productivity (Guiqing et al., 2024); On the other hand, moderate water deficit during the late stage of fruit enlargement and ripening can affect fruit sugar concentration and acidity by regulating osmotic pressure (Chen et al., 2022; Gómez-Bellot et al., 2024). Appropriate habitat stress is beneficial for plant growth and fruit quality improvement, but exceeding the water and salt tolerance threshold can cause damage to the crop itself (Tan et al., 2009; Yang et al., 2021).

Although scholars have conducted extensive research on the effects of irrigation methods and water and salt distribution on root growth, aboveground yield, and quality of fruit trees, the response of fruit yield and quality of saline alkali fruit trees to changes in water and salt within the main root range has not been fully understood. In addition, traditional simulations pursue the optimal water and salt distribution scheme, lacking verification from the perspective of economic benefits from production practice. The shortcomings of this theory may limit the comprehensive evaluation of the impact of drip irrigation strategies on the economic benefits of forests and fruits in saline alkali areas from the perspective of producers. To investigate the matching degree between irrigation decision-making schemes and salt alkali pear production, we focused on the salt alkali soil in southern Xinjiang, using the typical forest fruit Korla pear as the experimental object. Through two years of experiments, we explored: 1) using the HYDRUS-2D model to simulate the soil moisture and salt content in the root zone of Korla pear, and selected a simulation scheme suitable for surface drip irrigation of pear during the initial fruiting stage in arid salt alkali areas. 2) Using multi-objective optimization methods to scientifically quantify yield, quality, and net profit, explore irrigation strategies suitable for fragrant pears under the condition of maximum comprehensive economic benefits, and verify the accuracy of numerical simulation in production practice. The research results can provide a comprehensive evaluation perspective and technical support for optimizing surface drip irrigation technology and formulating irrigation strategies for saline alkali fruit trees.

## 2 Materials and methods

### 2.1 Experimental site description

**Figure 1:**
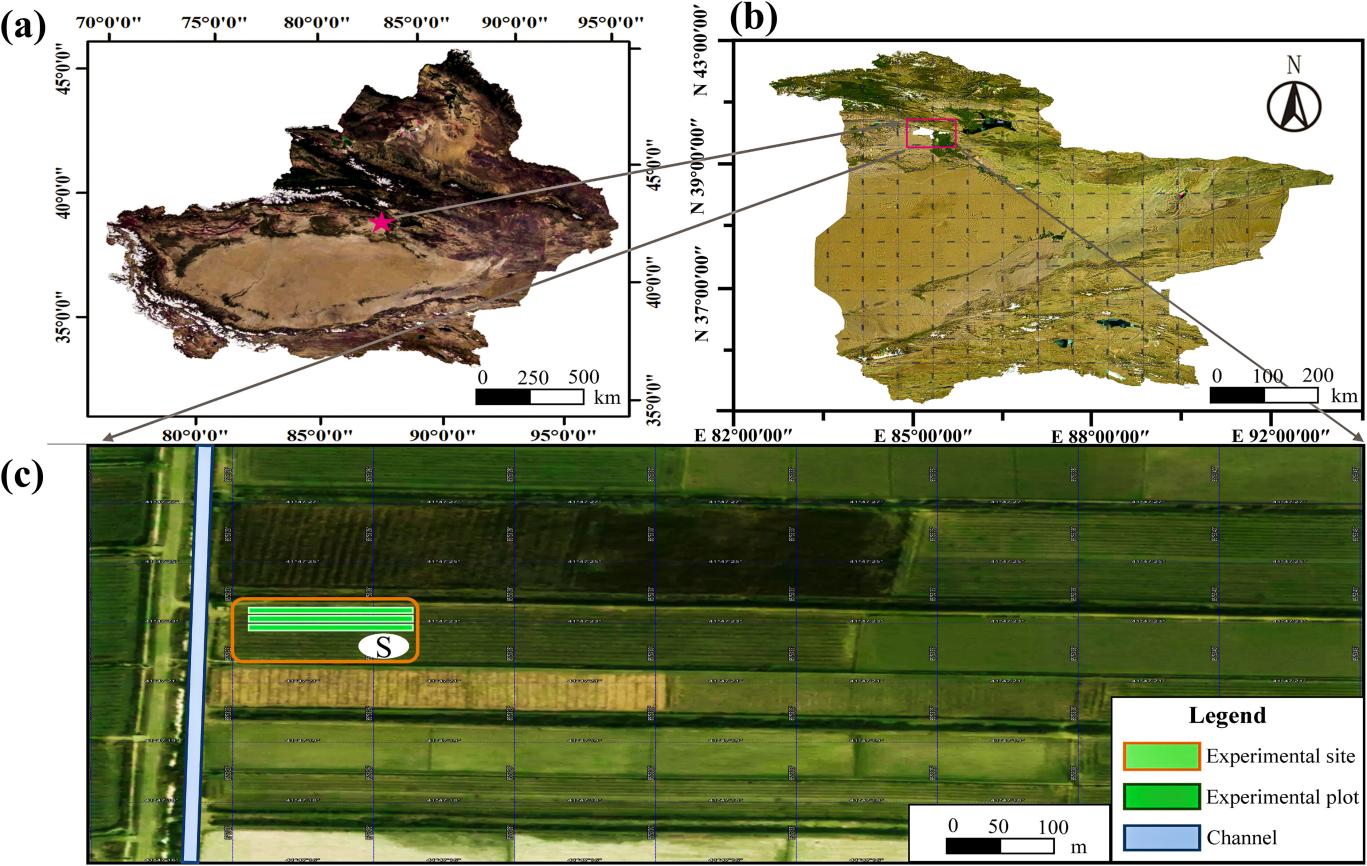
Location map of the experimental site. **(A)** Map of Xinjiang Uygur Autonomous Region.; **(B)** Map of Bayingolin Mongolian Autonomous Prefecture; **(C)** Aerial view of the experimental site.

**Figure 2:**
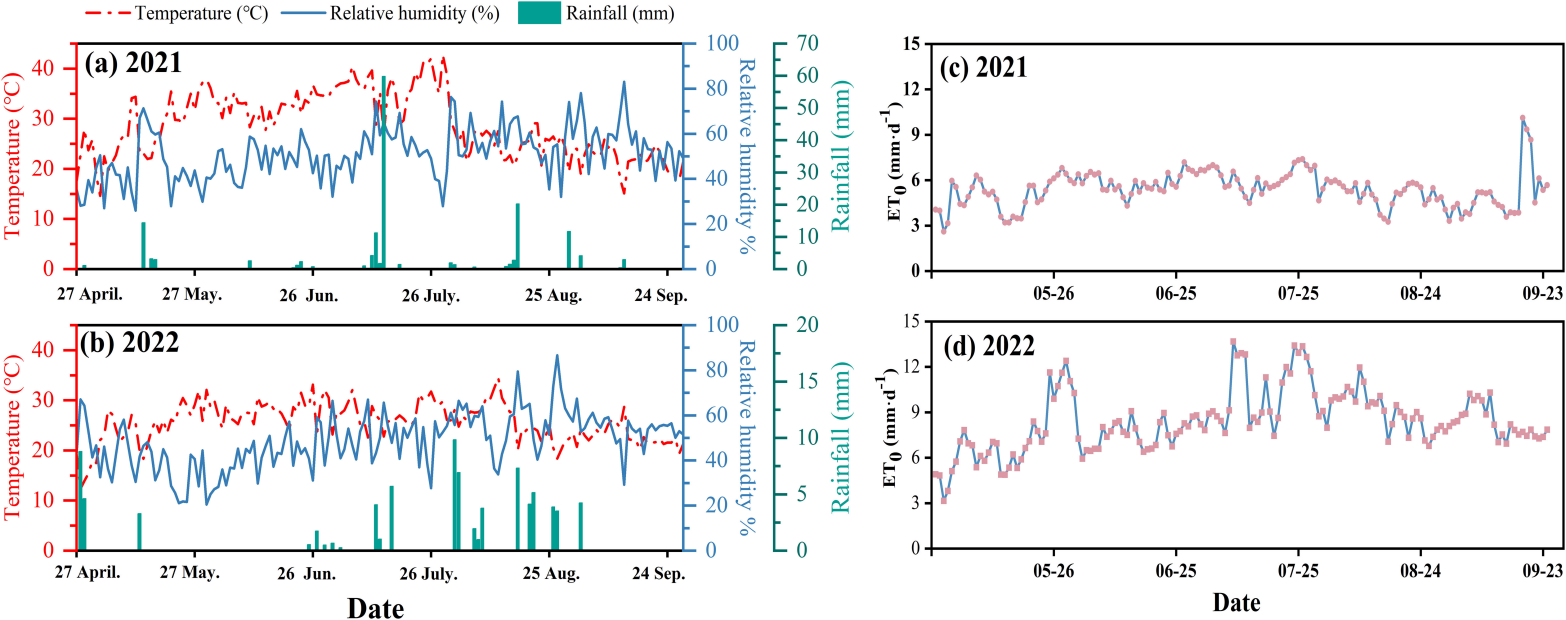
Meteorological data for the test area. **(A, B)** are temperature, rainfall and relative humidity during the reproductive period in 2021 and 2022. **(C, D)** are the dynamics of reference crop evapotranspiration (ET0) in April-September for 2021 and 2022.

The experiment was performed during the 2021–2022 fertility period (April–September) in a typical fragrant pear orchard in Korla, Xinjiang (85°53′E, 41°47′N, 910 m asl, **Figure 1**). The experimental area has a typical temperate continental climate, with an average annual temperature, annual rainfall, annual evaporation, total number of sunshine days, and average wind speed of 12.5°C, 108 mm, 2790 mm, 2990 h, and 2.18 m·s^−1^ (at 2 m), respectively. The experiment station was equipped with the Tianqi Intelligent Ecological Station (INSENTEK Oriental Zhigan Co., Ltd., Zhejiang, China) to monitor meteorological elements (**Figure 2**). The soil type at the 0–100 cm depth of the experimental area was sandy loam, with a soil organic matter content of 15.0 g·kg^−1^, total nitrogen of 0.6 g·kg^−1^, alkali-hydrolyzable nitrogen of 182.8 mg·kg^−1^, available potassium of 206.4 mg·kg^−1^, ammonium nitrogen of 5.0 mg·kg^−1^, and nitrate nitrogen of 17.2 mg·kg^−1^. Moreover, the pH was 8.2, the field water holding capacity of the soil was 19.16%, and the saturated water content was 27.79% (**Table 1**). The groundwater was buried at a depth of 9 m, with a conductivity of 90 μs·cm^−1^.

**Table 1:** Main physical properties of the 0-100 cm tillage layer in the experimental area.

Soil depth (cm)	Soil texture	Soil particle composition			pH value	Bulk Density (g·cm^-3^)	Soil salinity (g·kg^-1^)	Soil moisture		
		Sand (%)	Silt (%)	Clay (%)				Saturated Water Content (%)	Field Water Holding Capacity (%)	Wilting Point (%)
0-20	Sandy loam	27.30	68.49	4.21	8.16	1.47	8.76	24.58	18.62	7.03
20-40	Sandy loam	26.09	70.09	3.82	8.07	1.50	12.18	23.94	17.68	7.33
40-60	Sandy loam	50.65	46.40	2.95	8.06	1.39	12.33	25.79	19.08	7.65
60-80	Sandy loam	47.48	48.94	3.58	8.17	1.40	12.20	29.50	22.61	7.74
80-100	Silt loam	43.23	53.54	3.23	8.30	1.42	13.70	35.16	19.09	7.80

The content of soil particle size was determined by laser particle size analyzer (LS13320, Beckman Coulter, Shanghai, China), and the soil particles were graded according to the USDA Soil Taxonomy system of Soil Classification Standards(Millán et al., 2003).

### 2.2 Experimental design

**Figure 3:**
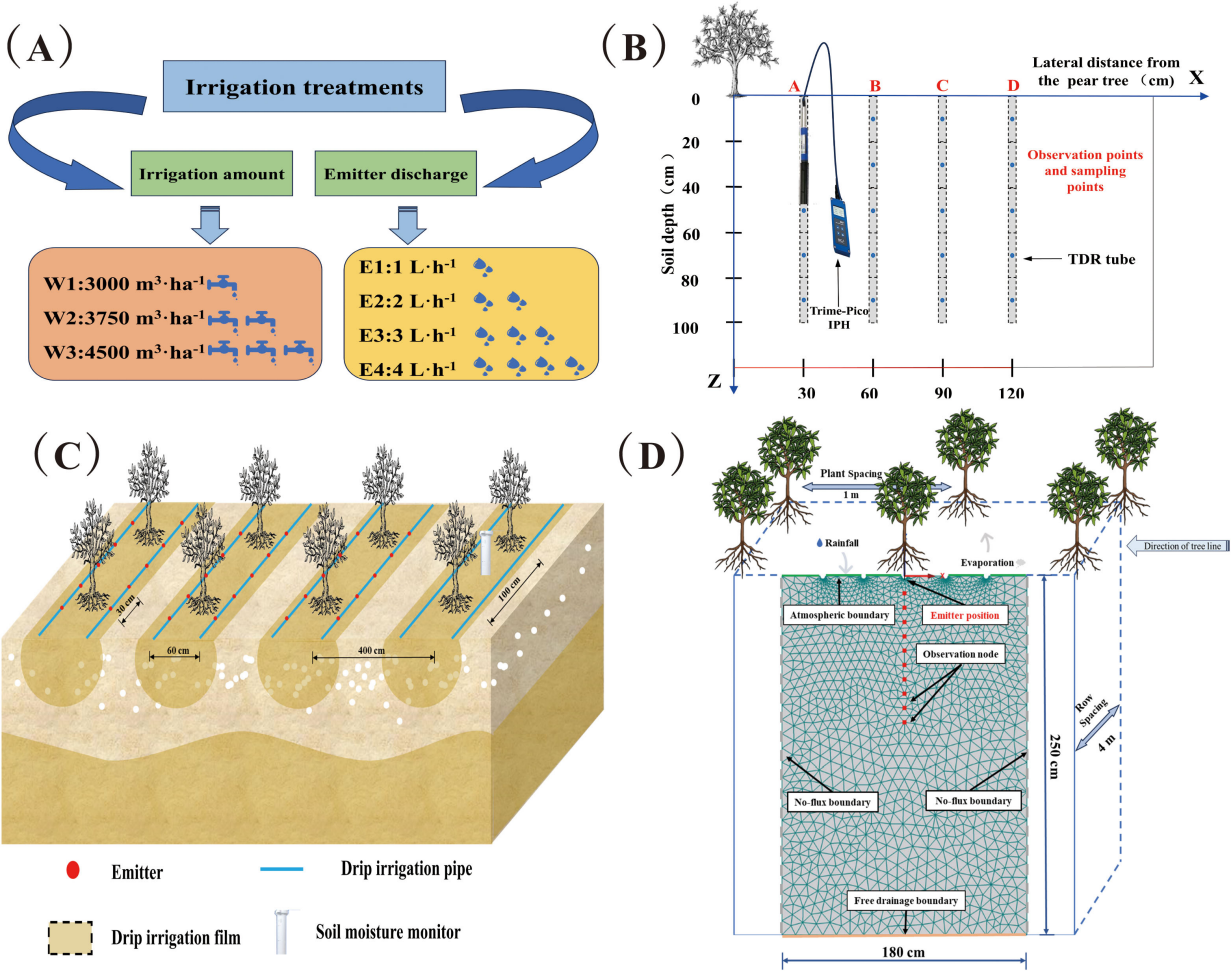
Schematic diagram of experimental design. **(A)** Schematic diagram of experimental treatments; **(B)** Schematic diagram of experimental sampling; **(C)** Schematic diagram of fragrant pear planting and drip irrigation tape deployment; **(D)** Schematic diagram of the HYDRUS-2D model boundary conditions in the experimental area.

The experiment was performed during April–September 2021 and 2022. We selected 6a-old Korla fragrant pear (*Pyrus sin-kiangensis yu*) as the test subject. The experiment included four emitter discharges (E1, 1 L·h^−1^; E2, 2 L·h^−1^; E3, 3 L·h^−1^; E4, 4 L·h^−1^), three irrigation quotas (W1, 3000 m^3^·ha^−1^; W2, 3750 m^3^·ha^−1^; W3, 4500 m^3^·ha^−1^, **Figure 3A**) to investigate the impact of these treatments on the experimental indices. Three replications were performed for each treatment The experimental plots had an area of 190 m^2^, with an average of 35 fragrant pear plants (4 (row spacing) × 1 (plant spacing)). Irrigation and fertilization were performed using surface drip irrigation, with two tubes in one row and a pressure-compensated emitter. The emitter spacing was set as 30 cm and drip tape was 30 cm away from the tree (**Figure 3C**). A total of 900 kg·ha^−1^ of fertilizer was applied throughout the reproductive period via dripping alongside the irrigation water (**Table 2**). In the pre-flowering period of fragrant pear, all treatments were applied to the base fertilizer. Winter irrigation salt washing (diffuse irrigation) was then carried out in late October for both years, totaling 3600 m^3^·ha^−1^. This was followed by spring irrigation (diffuse irrigation) at the end of March, with a quota of 1500 m^3^·ha^−1^. Standard uniform agronomic methods were employed throughout the reproductive periods of the fruit trees, guaranteeing consistency of management in the field Basic soil physical properties in the test area were collected and determined before the start of the experiment (**Table 1**).

**Table 2:** Irrigation and fertilization schedule of Korla fragrant pear in the experimental area.

Years	Number of irrigation	Irrigation date	Irrigation amount(m^3^·ha^-1^)			Fertilization amount (kg·ha^-1^)
			W1	W2	W3	
2021	1	20 April 2021	150	187.5	225	0
	2	05 May 2021	150	187.5	225	0
	3	20 May 2021	150	187.5	225	150
	4	05 June 2021	375	468.75	562.5	150
	5	20 June 2021	375	468.75	562.5	0
	6	05 July 2021	450	562.5	675	225
	7	21 July 2021	450	562.5	675	225
	8	05 August 2021	450	562.5	675	150
	9	25 August 2021	450	562.5	675	0
Total			3000	3750	4500	900
2022	1	21 April 2022	150	187.5	225	0
	2	07 May 2022	150	187.5	225	0
	3	22 May 2022	150	187.5	225	150
	4	07 June 2022	375	468.75	562.5	150
	5	22 June 2022	375	468.75	562.5	0
	6	08 July 2022	450	562.5	675	225
	7	23 July 2022	450	562.5	675	225
	8	09 August 2022	450	562.5	675	150
	9	28 August 2022	450	562.5	675	0
Total			3000	3750	4500	900

The first application was a balanced fertilizer, the second and third were high phosphorus, the fourth and fifth were high potash, and the N-P-K ratios of the three fertilizers were 20-20-20, 16-44-0 and 8-12-32.

### 2.3 Measurements

#### 2.3.1 Soil water and soil salinity contents

One fragrant pear tree was randomly selected from each treatment at the beginning of the experiment. TDR (time domain reflectometry) tubes were deployed at 0, 30, 60, and 90 cm horizontal distances perpendicular to the rows of the selected tree, using the trunk as the reference (**Figure 3B**). The Shang Crop Soil Monitor ET100 (INSENTEK Oriental Zhigan Co., Ltd., Zhejiang, China) was set up in the center between two trees along the tree row direction for the real-time determination of rainfall, temperature, and evapotranspiration. The TRIME-PICO IPH/T3 TDR soil moisture monitoring system (IMKO, Ettlingen, Germany) was used to measure the volumetric water content of the soil at 20 cm intervals vertically from the 0–100 cm soil depth. The gravimetric method was used to verify the moisture probes once every 15 days. Measurements were taken before and after each irrigation event during the pear reproductive period, as well as before and after rainfall events. Furthermore, sampling points were set up at 30 cm intervals from 0–90 cm horizontally perpendicular to the direction of the tree rows within 24 h before and after each irrigation. Soil samples were collected from the 0–100 cm soil profile using five soil augers positioned at 20 cm intervals. To minimize experimental errors, three replications were set up for each treatment and the average of the three sets of the results was selected for the subsequent analysis. The soil electric conductivity (EC1:5) and soil salinity (S) per unit area of soil (1 m^2^) were determined using a conductivity meter (FE38−Standard, Mettler Toledo, Shanghai, China). The EC value of the soil solution simulated by HYDRUS-2D was converted to soil salinity S as follows:


(1) $$S=0.0033EC_{1:5}+1.9026,R^{2}=0.98$$

where *S* is the soil salinity (g·kg^−1^); and EC_1:5_ is the electrical conductivity of the soil leachate (μs·cm^−1^) (Guo et al., 2015).

#### 2.3.2 Yield and quality measurements

During the ripening period, three fragrant pear trees were randomly selected from each treatment in the east, south, west, north, and center, with 3–5 fruit samples from each treatment. The single fruit weight (F_w_, g) was measured using an MP200 electronic balance and soluble solids were determined using a hand-held saccharimeter. Each measurement was repeated three times and averaged. Three additional fragrant pear trees were randomly selected from each treatment and the mass of all fragrant pears on each pear tree was weighed separately. The average value was taken as the single plant yield of the treatment. According to the national standard GB/T 19859-2005 of the People’s Republic of China (Wang et al., 2024), the yield (Y, kg·ha^−1^) of each treatment was calculated as:


(2) Y=n×M

where *Y* is the fragrant pear yield (kg·ha^−1^); *n* is the number of fragrant pear tree plants in the treatment plot (plants·ha^−1^); and *M* is the yield of a single plant (kg·plant^−1^). The net post-harvest profit of fragrant pear was described as:


(3) $$N=G-W_{C}-F_{C}-L$$

where *N* is net profit (RMB·ha^−1^); *G* is economic income (RMB·ha^−1^) and is calculated via equation (4); *W_C_* (RMB·ha^−1^) is the utility cost for the entire reproductive period; *F_C_* is the fertilizer cost (RMB·ha^−1^); and *L* (RMB·ha^−1^) denotes other costs (**Supplementary Table S3**).


(4) G=11a+7.9b+6c

where *a*, *b*, and *c* denote the mass (kg·ha^−1^) of A, B, and C grade fruits per hectare, respectively. Korla fragrant pear fruit of grades A, B, and C with a purchase guide of 11, 7.9 and 6 RMB·kg^−1^ (Zhang et al., 2022a; Zhang et al., 2022b).

### 2.4 HYDRUS-2D model simulation

HYDRUS-2D (Šimůnek et al., 2008) has been proven to perform well in two-dimensional soil water balance simulations and is widely used to solve Richards’ equation (Jacques et al., 2008) based on finite element grid numerical solutions.

#### 2.4.1 Evapotranspiration

Reference crop evapotranspiration (*ET*_0_) was calculated using the Penmen–Monteith equation recommended by FAO-56 (Pereira et al., 2015), expressed as:


(5) $$ET_{0}=\frac{0.408\text{Δ}(R_{n}-G)+\gamma \frac{900}{T+273}U_{2}(e_{a}-e_{d})}{\text{Δ}+\gamma (1+0.34U_{2})}$$

where *ET*_0_ is the reference evapotranspiration (mm·d^−1^); *R_n_* is the net solar radiation (MJ·m^−2^·d^−1^); *G* is the soil heat flux density (MJ·m^−2^·d^−1^); *T* is the daily mean air temperature at a 2 m altitude (°C); *U*_2_ is the wind speed at a 2 m altitude (m·s^−1^); *e_a_* is the saturated vapor pressure (KPa); *e_d_* is the actual vapor pressure (KPa); and *γ* is the humidity constant (KPa·°C^−1^).

The potential evapotranspiration (ETp) is calculated as follows:


(6) $$ET_{p}=KcET_{0}$$

where Kc is the crop coefficient, which equals 1.12 during florescence (0–25 days after flowering); 1.12–1.21 during the fruit setting period (26–59 days after flowering), 1.84 during the fruit swelling period (60–112 days after flowering), and 1.45–1.84 during the mature period (113–134 days after flowering) (Zhang et al., 2022b).

Tp and Ep can be calculated from ETp by Beer’s law (Yang et al., 2019) as follows:


(7) $$T_{p}(t)=ET_{p}(t)[1-\exp(-\eta LAI)]$$

(8) $$E_{p}(t)=ET_{p}(t)-T_{p}(t)$$

where LAI is the leaf area index; and η is the extinction coefficient (a general empirical factor of 0.39), which LAI was set as 2.57 in this study. Tp (mm·d^−1^) and Ep (mm·d^−1^) were used as input values for the boundary conditions in the HYDRUS-2D model.

#### 2.4.2 Soil water flow

Richards’ equation was employed for the numerical models of the soil water flow as follows:


(9) $$\frac{\partial \theta (h)}{\partial t}=\frac{\partial }{\partial x}\left[K(h)\frac{\partial h}{\partial x}\right]+\frac{\partial }{\partial x}\left[K(h)\frac{\partial h}{\partial z}+K(h)\right]-S(x,z,\text{h})$$

where θ(h) is the volumetric soil water content (SWC) (cm^3^·cm^−3^); h is the pressure head (cm); K(h) is the unsaturated hydraulic conductivity of the soil (cm·d^−1^); t is the time (T) (d); x is the horizontal coordinate (L); z is the vertical coordinate (L); and S(h) is the root water uptake (RWU) in the source–sink term, cm^3^·(cm^3^·d) ^−1^ (Feddes et al., 1976), defined as follows:


(10) $$S(h)=\alpha (h)S_{p}$$

where S_p_ is the water uptake rate during the no-water stress cycle at α(h) = 1; and α(h) is the dimensionless response function of water uptake by the plant root system (Hartmann et al., 2018), and is described as follows:


(11) $$\alpha (h)=\left\{ \begin{array}{l} \frac{h_{1}-h}{h_{1}-h_{2}}\;h_{2}<h\leq h_{1}\\ \frac{h-h_{4}}{h_{3}-h_{4}}\;h_{4}\leq h\leq h_{3}\\ \;1\;h_{3}\leq h\leq h_{2}\\ \;0\text{ other} \end{array}\right.$$

where h_1_, h_2_, h_3_, and h_4_ are water matrix potential values (h_1_ =-10 cm; h_2_=-25 cm; h_3_=-50 m; and h_4_=-80 m). The soil moisture characteristic curve and unsaturated hydraulic conductivity in equation (5) were described using the van Genuchten–Mualem equation (Van Genuchten and Wagenet, 1989) as follows:


(12) $$\theta (h)=\left\{ \begin{array}{l} \theta_{r}+\frac{\theta_{s}-\theta_{r}}{{\left[1+{\left(\alpha \left|h\right|\right)}^{n}\right]}^{m}}\;h<0\\ \theta_{s}\;h\geq 0 \end{array}\right.$$

(13) $$K(h)=K_{\text{s}}{\text{S}}_{e}^{l}\;{\left[1-{\left(1-S_{e}^{l/m}\right)}^{m}\right]}^{2}$$

(14) $$S_{e}=\frac{\theta (h)-\theta_{r}}{\theta_{s}-\theta_{r}}$$

where θ_r_ is the residual water content (cm^3^·cm^−3^); θ_s_ is the saturated water content (cm^3^·cm^−3^); Ks is the saturated hydraulic conductivity of the soil (cm·d^−1^); Se is the effective saturation (-); α (cm^−1^), n (-), and m (-) are empirical parameters; and l is the soil pore connectivity parameter.

#### 2.4.3 Root water uptake

According to the Feddes mode (Feddes et al., 1976) in HYDRUS-2D, the water stress response function can be determined from the parameters in the HYDRUS database (**Supplementary Table S1**). Salt stress was estimated using a threshold model, with a threshold and slope of 3.4 ds·m^−1^ and 12, respectively (Burrows and Stott, 1999; Grattan, 2016; Tanji et al., 2002) and maximum and minimum root depth of 150 cm and 20 cm, respectively. The water stress response function was determined as follows:


(15) $$S(h,h_{\varphi },x,z)=\alpha (h,h_{\varphi },x,z)b(x,z)S_{t}T_{p}$$

(16) $$\int_{\Omega_{R}}b(x,z)d\Omega =1$$

where S is the root water uptake term; α(h, hφ, x, z) is the soil water and salt stress function; hφ is the osmotic pressure (cm); b(x, z) is the root distribution function (cm^−2^); St is the width of the surface in terms of transpiration (cm); Tp is the potential crop transpiration (cm·d^−1^); R is the radius of the root water uptake area (cm); and ΩR is the root distribution area (cm^2^).

#### 2.4.4 Soil solute transport model

The basic equation for salt transport is described as follows (Li Y. et al., 2023):


(17) $$\frac{\partial (\theta )}{\partial t}=\frac{\partial }{\partial x_{i}}(\theta D_{ij}\frac{\partial c}{\partial x_{i}})-\frac{\partial (q_{i}c)}{\partial x_{i}}-S(h)C_{s}$$

where c is the solute concentration (g·cm^−3^); qi is the flux (cm·d^−1^); Dij is the dispersion coefficient (cm^2^·d^−1^); subscripts i and j denote the x- and z-axis coordinates, respectively; and Cs is the salt content of the soil sinks (g·L^−1^).

#### 2.4.5 Initial and boundary conditions

The vertical plane XZ, with dimensions 250 (height) × 180 (width) cm, was selected for the simulation area. A total of 10 observation nodes were set every 10 cm below the emitter (**Figure 3D**). The 2021 and 2022 fertilities were set as the calibration and validation simulation times, respectively. A finite element (FE)-mesh unstructured generation model was employed to simulate the experimental treatments. The emitters were represented by 1 cm-diameter half-circles on the top boundary and set as variable flux boundaries. The other areas on the top boundary were set as atmospheric boundaries, Input rainfall data collected by meteorological stations as meteorological conditions into the model, while the left and right boundaries were taken as no-flux boundaries. During irrigation, the flux of the drip emitters was described in HYDRUS-2D as follows:


(18) $$Q=\frac{q}{L\times 2\pi R}$$

where Q is the input water flux of an individual emitter (cm·day^−1^); R is the radius of the emitter (0.5 cm); and L is the emitter spacing between the drip emitters (30 cm).

#### 2.4.6 Model parameters

The soil moisture parameters were estimated using the Rosetta module (Šimůnek and Suarez, 1993) in HYDRUS (2D/3D) based on the soil bulk weight and soil grain size composition determined before the experiment (θr, θs, Ks, α, n, and l) and calibrated soil hydraulic parameters (**Table 3**). The Van Genuchten–Mualem equation (Van Genuchten and Wagenet, 1989) was adopted as the soil moisture characteristic function of the model and the parameters were corrected using measured data. The ET flux input into the model was derived from the continuous observational data of the intelligent soil water and salt monitoring system. The hydrodynamic dispersion coefficient was determined from the breakthrough curve (BTC), where the longitudinal dispersivity (DispL) of the solute transport parameter was set to 20 cm and the transverse dispersivity (DispT) to 4 cm.

**Table 3:** Basic physical properties of soils and calibrated model parameters.

Soil depth/cm	Model parameters/Calibrated model parameters					
	*θ*_r/(_cm^3·^cm^-3)^	*θ*_s/(_cm^3·^cm^-3)^	*K*_s/(_cm·d^-1)^	*α/*_(_cm^-1^)_·_	*n*(-)	*l*(-)
0-20	0.038/0.040	0.36/0.39	42.75/42.93	0.01	1.60/1.63	0.5
20-40	0.040/0.042	0.35/0.38	39.63/40.77	0.01	1.58/1.60	0.5
40-60	0.030/0.031	0.37/0.38	73.36/73.11	0.01	1.48/1.47	0.5
60-80	0.030/0.030	0.35/0.35	50.78/50.76	0.01	1.48/1.48	0.5
80-100	0.030/0.030	0.35/0.35	47.17/47.08	0.01	1.50/1.47	0.5

The soil physical property index was the average value of the study area, where *θ_r_* is residual water content; *θs* is saturated water content; *Ks* is saturated hydraulic conductivity; *α* is shape factor; *n* is empirical parameter; *l* is pore correlation.

#### 2.4.7 Calibration and validation

Numerical simulations were performed using the experimental data collected in 2021 and 2022. The soil parameters were calibrated and corrected with the 2021 data (**Table 3**) and were then used for the 2022 model simulation. To quantify the accuracy of the HYDRUS-2D model, the reliability of the simulation results was assessed using the root mean squared error (RMSE) and Nash–Sutcliffe efficiency coefficient (NSE) (Yao et al., 2023) as follows:


(19) $$RMSE=\sqrt{\frac{1}{n}\sum_{i=1}^{n}{\left(S_{i}-O_{i}\right)}^{2}}$$

(20) $$NSE=1-\frac{\sum_{i=1}^{n}{\left(S_{i}-O_{i}\right)}^{2}}{\sum_{i=1}^{n}{\left(O_{i}-\bar{O}\right)}^{2}}$$

where S_i_ is the simulated value; O_i_ is the measured value; $\overset{-}{O}$ is the average of the measured values; and n is the number of samples.

### 2.5 Comprehensive evaluation of economic benefits

To verify the accuracy and rationality of simulation results in production practice, the boundary conditions for multi-objective solution are determined using actual output, quality, and economic benefits. Due to the different dimensions of production, physical/flavor quality, and net profit, each evaluation indicator is linearly normalized. Define acceptable regions as relative values ≥ 70%, ≥ 80%, and ≥ 90%, respectively. Then, based on spatial analysis, project the contour lines of each evaluation indicator onto a plane, and the intersection area of the contour lines represents the interval that simultaneously satisfies the evaluation indicators.

### 2.6 Data analysis

Experimental data were processed using Excel 2021 (Microsoft Corp.) and plotted using Origin 2021 (Origin Lab, Northampton, MA, USA). Significance differences were assessed using the least significant difference (LSD) method and analysis of variance (ANOVA) was performed using SPSS 26.0 (SPSS Inc., IL, USA).

## 3 Results

### 3.1 Model evaluation

**Figure 4:**
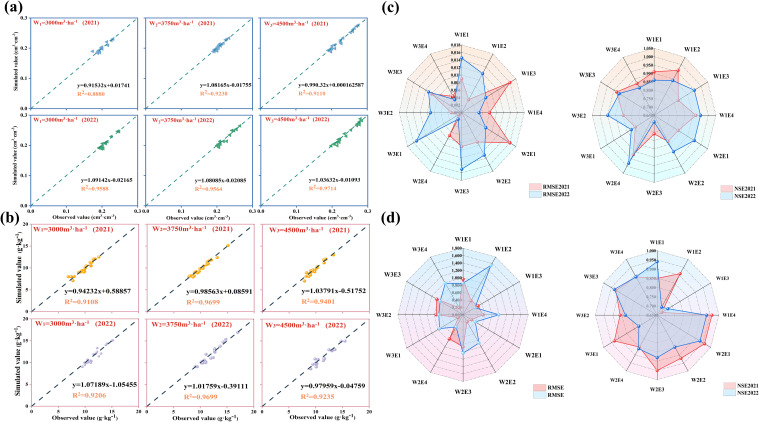
Consistency test between simulated and measured values. **(A, B)** Regression equations between measured and simulated values for different irrigation conditions in 2021 and 2022.The fitting curves are statistically significant (P< 0.01). The dash represents 1:1 of the measured value and the simulated value. (blue border: SWC; red border: SSC). **(C, D)** Changes in statistical indicators RMSE and NSE for different treatments SWC and SSC in 2021 and 2022.

The HYDRUS (2D/3D) model was calibrated and validated using measured data collected in 2021 and 2022, respectively. **Figures 4A, B** resents the coefficient of determination (R^2^) and the linear regression equation between the modelled and measured SWC and SSC at the 0–100 cm soil depth in 2021 and 2022. The R^2^ values of SWC and SSC under different irrigation treatments in 2021 and 2022 were 0.89, 0.92, 0.91and 0.96, 0.96, 0.97 in 2021; 0.91, 0.97, 0.94 and 0.92, 0.97; 0.92 in 2022, respectively. The RMSE and NSE were used to compare the consistency between the simulated and measured SWC and SSC values under different treatments (**Figures 4C, D**). The RMSE and NSE of SWC (SSC) reached RMSE:0.02–0.16 cm^3^·cm^−3^ (0.22–1.54 g·kg^−1^), and NSE:0.76–0.95 (0.68–0.96), respectively. At the same irrigation amount, the emitter discharge 4 L·h^−1^ exhibited less volatility in terms of the RMSE and NSE values compared to 1 L·h^−1^, and the measured values were in better agreement with the modelled values (**Supplementary Table S2**). In addition, the R^2^ > 0.85 and NSE > 0.75 indicate that the model provides a good estimation of soil water content and salinity. Therefore, the subsequent simulations and analyses were carried out with the 2022 corrected parameters for the three irrigation levels at 4 L·h^−1^.

### 3.2 Simulation of soil water and SSC dynamics in 2022

**Figure 5:**
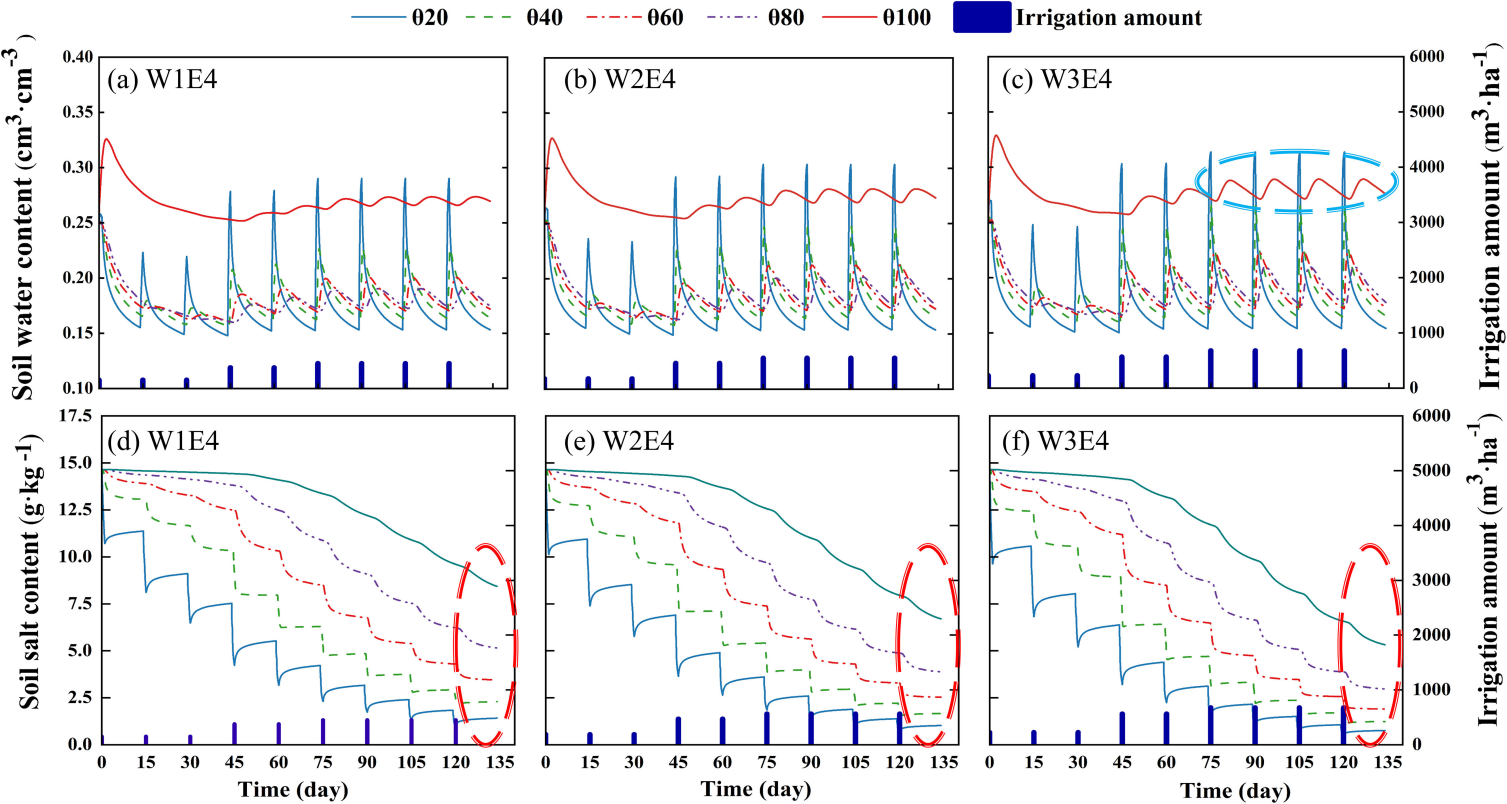
Changes in water-salt dynamics during the reproductive period of fragrant pear. **(A–C)** is 0-100 cm SWC change; **(D, E)** is 0-100 cm SSC change. Where **(A, D)** are W1E4 treatments; **(B, E)** are W2E4 treatments; **(C, F)** are W3E4 treatments.

The dynamics of SWC and SSC at different soil depths at three irrigation amounts (W1, W2, and W3) were modelled over the complete fertility period (**Figure 5**). The results showed that the SWC of each treatment exhibited a ‘sawtooth’ trend, whereby values decreased gradually until the next irrigation and subsequently increased. Moreover, the fluctuation range of SWC was larger in the middle and late stages compared with the early stage, depending on the amount of water used in a single irrigation. In terms of soil depth, the SWC exhibited a reduction in fluctuations with increasing soil depth, and the difference in the change of SWC in the 20 cm depth range decreased with increasing irrigation volume, while the SWC in the 40 cm-100 cm depth gradually increased (**Figures 5A–C**). In particular, the SWC range at the 100 cm soil depth exhibited the following trend: W3 > W2 > W1. This indicates that enhancing the irrigation amount facilitated the deeper infiltration of and greater SWC in the range of the main root system (0–80 cm). This is beneficial for ensuring the water supply and root water absorption strength of the main effective root zone (0-80 cm), and can promote the growth and development of dense root layer roots and aboveground parts. Before the onset of the reproductive period, the salts in the soil profile showed subsoil aggregation in the 80–100 cm layer (**Table 1**), which was gradually washed downwards after the irrigation event. The salinity in the 0–20 cm surface soil exhibited a decreasing and subsequently increasing trend due to temperature and evapotranspiration conditions, and was reduced by an average of 81.4–86.1% after irrigation throughout the reproductive period. The relationship between irrigation treatments and salinity reduction was as follows: W3 > W2 > W1. In addition, the increase in irrigation amount effectively promoted salt-leaching from the deep soil, and the salinity at 100 cm depth was reduced by an average of 21.30% at the same emitter discharge in the W3 treatment compared with the W1 treatment (from the beginning to the end of the reproductive period). Thus, the increase in the irrigation amount promotes the migration of soil moisture to the deeper soil, which in turn strengthens the salt-leaching effect of the deeper soil and ensures the water–salt balance in the main root zone. This is conducive to the physiological growth of the root system of saline–alkali fruit trees.

### 3.3 Optimal irrigation amount and emitter discharge treatment based on multi-objective optimization

Irrigation treatments can reduce the inhibitory effect of inter-root soil salinity on the root system and the upper plant components. Moreover, maintaining a reasonable water–salt environment is beneficial to the root system of fruit trees, while the response of above-ground fruit yield and quality is the basis for assessing the effectiveness of irrigation. The irrigation amount and emitter discharge treatments significantly (*p*< 0.01) affected the yield and quality indexes (i.e., fruit weight and soluble solid) of fragrant pear. Yield increased with the irrigation amount at the same emitter discharge treatment (W3 > W2 > W1), within W3 exhibiting an average increase of 10.55% and 24.60% over W2 and W1 treatments, respectively (**Table 4**). Fruit weight the physical quality of fruit increased and then decreased as the emitter discharge increased and were positively correlated with the irrigation amount. Under W3, fruit weight was observed to be higher in the E3 treatment by 9.05%, 16.25%, and 19.40% compared to treatments E4, E2, and E2 respectively (**Table 4**). Irrigation and emitter discharge had a significant (*p<* 0.01) impact on soluble solids, an indicator of the chemical quality of fruits, with an overall positive correlation between irrigation amount and soluble solids. However, the inter-annual variations were not significant in 2021 and 2022, and the rate of increase slowed down in 2022. The incremental rates from E1 to E4 under W3 in 2021 (2022) were 16.38%, 9.38%, and 5.44% (5.88%, 4.58%, and 3.13%) (**Supplementary Figure S1C**). Water and electricity, fertilizer, and other costs (WC, Fc, and L) exhibited limited variations between the two years (**Supplementary Table S3**). Thus, net profits are influenced by yield and the overall trend in net profits is in line with yield (**Supplementary Figure S1D**).

**Table 4:** Effect of different irrigation treatments on yield, quality and overall economic efficiency.

Treatment	Properties (2021a)				Properties (2022a)			
	Yield (kg ha^-1^)	Fruit weight(g)	Soluble solid (%)	Net profits (RMB ha^-1^)	Yield (kg ha^-1^)	Fruit weight(g)	Soluble solid (%)	Net profits (RMB ha^-1^)
W1E1	9055.81 ± 79.35Cd	103.43 ± 0.50Cd	10.67 ± 0.58Cc	38808.99 ± 382.48Cd	10266.78 ± 374.48Cd	118.94 ± 0.57Cd	10.39 ± 0.17Cd	48723.00 ± 1941.32Cd
W1E2	9479.161 ± 186.18Cc	116.89 ± 0.18Cc	10.90 ± 0.12Cc	40849.57 ± 897.41Cc	11529.76 ± 150.43Cc	134.42 ± 0.21Cc	10.86 ± 0.12Cc	55270.28 ± 963.38Cc
W1E3	9711.85 ± 190.45Cb	137.06 ± 0.43Ca	11.10 ± 0.17Cb	41275.46 ± 526.03Cb	11776.22 ± 185.84Cb	157.63 ± 0.50Ca	11.64 ± 0.14Cb	56547.94 ± 779.85Cb
W1E4	10058.92 ± 37.39Ca	124.05 ± 0.15Cb	11.81 ± 0.14Ca	42572.31 ± 327.22Ca	12761.64 ± 87.12Ca	142.66 ± 0.49Cb	11.91 ± 0.10Ca	61656.36 ± 451.65Ca
W2E1	9936.21 ± 135.68Bd	120.84 ± 0.19Bd	10.93 ± 0.15Bc	42527.56 ± 653.96Bd	11742.80 ± 90.06Bd	138.96 ± 0.22Bd	11.32 ± 0.10Bd	55496.25 ± 1516.36Bd
W2E2	10045.77 ± 129.43Bc	151.60 ± 1.41Bc	11.53 ± 0.42Bc	43055.63 ± 623.86Bc	12007.96 ± 140.23Bc	158.01 ± 0.61Bc	12.00 ± 0.14Bc	56237.80 ± 1369.02Bc
W2E3	11539.53 ± 19.31Bb	169.43 ± 0.90Ba	12.07 ± 0.12Bb	47363.52 ± 293.07Bb	13481.80 ± 309.45Bb	184.29 ± 0.72Ba	12.34 ± 0.16Bb	58787.26 ± 933.70Bb
W2E4	13038.97 ± 32.46Ba	162.95 ± 0.13Bb	12.70 ± 0.12Ba	52180.82 ± 808.47Ba	14357.60 ± 28.31Ba	175.89 ± 0.14Bb	12.72 ± 0.37Ba	63559.73 ± 1556.09Ba
W3E1	10743.94 ± 80.66Ad	132.30 ± 0.22Ad	11.23 ± 0.86Ac	45895.81 ± 388.77Ad	12308.27 ± 276.23Ad	152.15 ± 0.25Ad	12.32 ± 0.50Ad	58260.90 ± 1423.59Ad
W3E2	11538.08 ± 227.28Ac	157.40 ± 0.46Ac	12.17 ± 1.04Ac	49723.53 ± 1095.50Ac	12421.84 ± 224.45Ac	174.34 ± 0.53Ac	12.68 ± 0.27Ac	59635.21 ± 1410.50Ac
W3E3	12873.84 ± 80.96Ab	177.87 ± 1.51Aa	12.71 ± 0.30Ab	51341.91 ± 390.21Ab	14744.00 ± 64.80Ab	191.87 ± 1.04Aa	12.79 ± 0.17Ab	64640.02 ± 885.66Ab
W3E4	13754.89 ± 40.42Aa	170.56 ± 0.63Ab	13.43 ± 0.21Aa	56873.92 ± 126.85Aa	15376.97 ± 211.68Aa	179.45 ± 0.72Ab	13.10 ± 0.15Aa	68908.48 ± 634.40Aa
Source of variance								
Irrigation amounts(W)	**	**	**	**	**	**	**	**
Emitter(E)	**	**	**	**	**	**	**	**
W×E	*	*	*	*	*	*	*	*

Capital letters represent differences between different irrigation amounts, while lowercase letters represent differences between different drip head flow rates. Asterisks * indicate the level of significance: * *p*< 0.05, ** *p*< 0.01.

**Figure 6:**
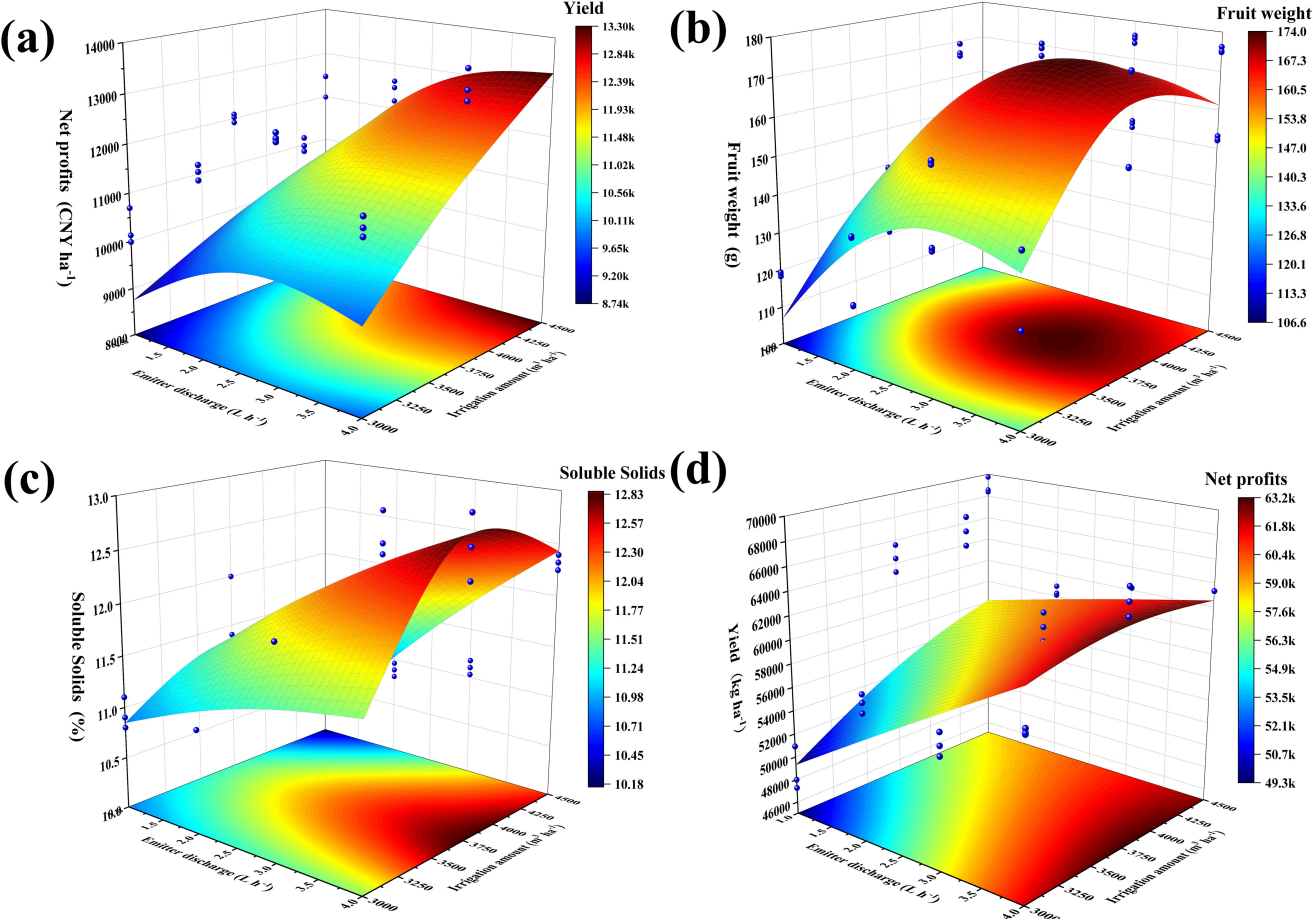
Three-dimensional surface plots corresponding to the level of irrigation amount and emitter discharge for each evaluation indicator. **(A)** fruit yield; **(B)** fruit weight; **(C)** soluble solids; **(D)** net profits.

**Figure 7:**
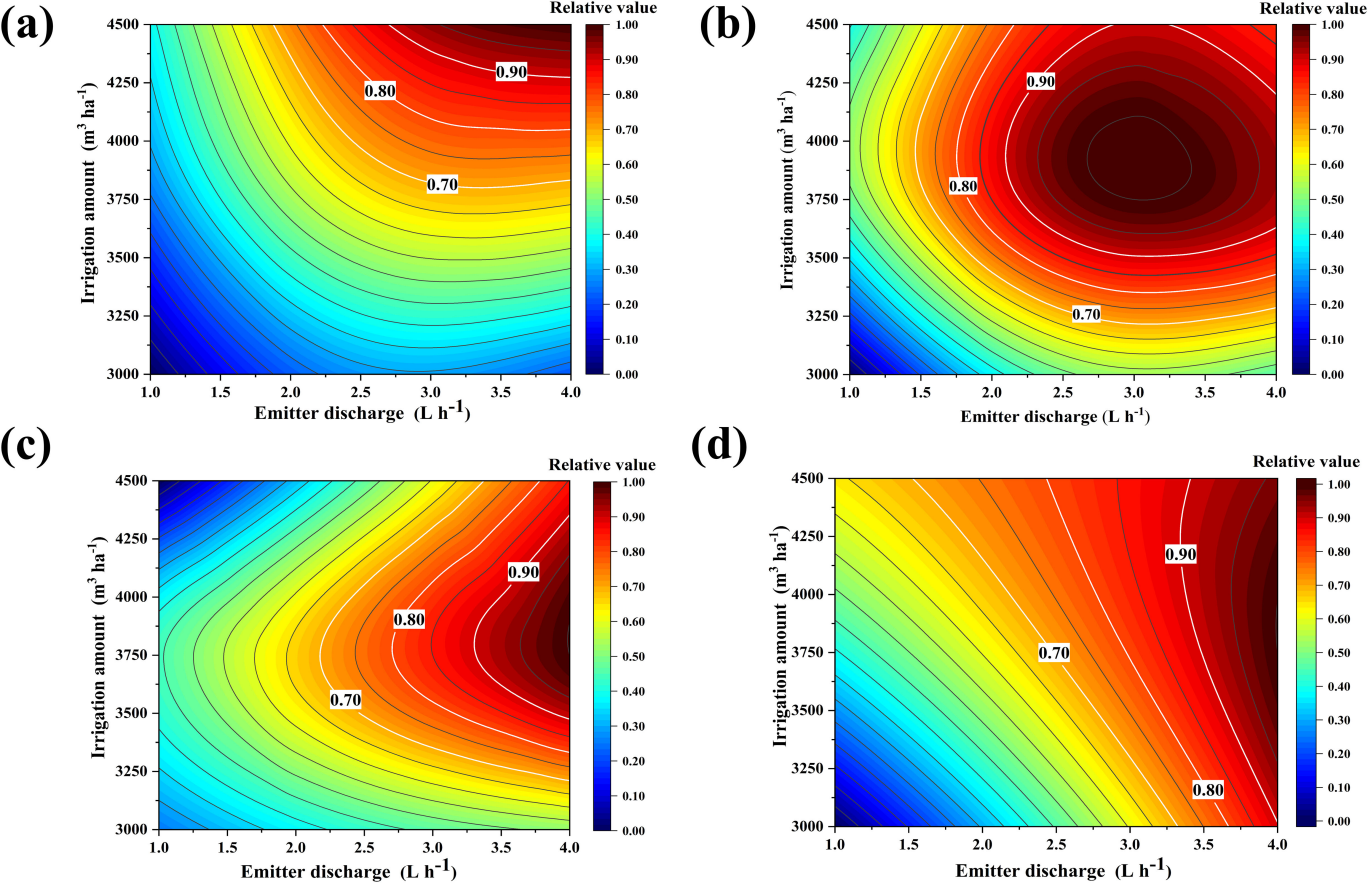
Response surface of the relative values of each evaluation in relation to irrigation amount and emitter discharge. **(A)** fruit yield; **(B)** fruit weight; **(C)** soluble solids; **(D)** net profits.

Fruit growers are not guided by a precise irrigation management model in actual agricultural practices and often believe that consistently increasing irrigation is the only way to reduce yield risk. However, the results of this study indicate that a mismatch between the irrigation strategy and root zone water salinity environment can affect yield and quality. To accurately quantify the yield, quality, and economic benefits, we set up a binary quadratic regression equation (**Table 5**) with the irrigation amount and emitter discharge as the independent variables, and yield, single fruit weight, soluble solids, and net profit as the dependent variables. This equation is used to investigate the optimal irrigation volume and emitter discharge when the above indexes reach their maximum values. The results revealed that the experimental treatments had different degrees of influence on the indicators, and the unit dimension of each evaluation indicator varied. Thus, it was not possible to use a single indicator as an evaluation criterion (**Figure 6**). To perform a comprehensive evaluation, we linearly normalized the data of the above indicators and deflated the data of each indicator according to the interval (0,1) (**Figure 7**). The regions with maximum values ≥90%, ≥80%, and ≥70% for each evaluation indicator are defined as acceptable regions, namely, the boundaries of these acceptable regions correspond to the 0.90, 0.80, and 0.70 contours in **Figure 7**. Based on the spatial analysis method, the contour of each of the above indicators was projected onto the plane to obtain a comprehensive evaluation and analysis map. The area of the region encircled by ≥70% to ≥90% was gradually reduced, yet the area corresponding to ≥70% and ≥80% in the circle was too large and connected with the coordinate axis. This led to a deviation from the extreme value. Thus, the overlap of the acceptable area corresponding to values ≥0.90 for each evaluation indicator was taken as an ideal range for satisfying the evaluation requirements. At the irrigation range of 4274–4297 m^3^·ha^−1^ and emitter discharge range of 3.82–3.88 L·h^−1^, the fragrant pear yield, fruit weight, soluble solids, and net profits could reach more than 90% of their maximum value.

**Table 5:** Regression modelling between irrigation amount and emission discharge and evaluation indicators.

Variables η	Regression Equation	R^2^	*p*
Yield	*η_1_* =-922.1 + 806.7E+3.932W+337.9E^2^+0.4039WE-0.0004232W^2^	0.91	<0.05
Fruit weight	*η_2_* =-432.8 + 47.9E+0.2661W-7.277E^2^-0.0004468EW-3.311×10^-5^W^2^	0.85	<0.05
Soluble solids	*η_3_* =-7.026-0.5659E+0.01032W-0.04754E^2^+0.000345EW-1.481×10^-6^W^2^	0.98	<0.05
Net profits	*η_4_* =-2329 + 9714E+21.16W-50.44E^2^-1.777WE-0.001798W^2^	0.80	<0.05

## 4 Discussion

### 4.1 Changes in soil moisture in the root zone under different irrigation treatments

The spatial distribution characteristics of the crop root system determine the extent of the action surface between the vegetation and the soil environment. Thus, the first step in developing an irrigation system is to match the soil moisture content with the water supply to the root zone, namely, the spatial co-ordination of root growth and the soil moisture distribution should be the goal of irrigation (Bughici et al., 2022; Wang et al., 2023). Previous studies have demonstrated that increasing irrigation quotas and enhancing deep soil moisture can effectively maintain water uniformity in the shallow soil profile (Yang et al., 2024). The distribution of the dense root layer of fruit trees is mainly concentrated in the 0–100 cm soil range (Li H. et al., 2024) In this study, the high irrigation treatment W3 was able to maintain a certain level of SWC, even at the 80–100 cm soil depth, while the W1 and W2 treatments were less effective in rehydrating water in deeper soils (**Figures 5A–C**). Water content within the longitudinal depth of the soil increased with the irrigation amount, which matched the distribution extent of the fruit tree fine roots. Numerous studies have shown that the emitter discharge is directly related to the characteristics of the wet zone in the root zone, and the higher the emitter discharge, the wider and shallower the soil wet zone, and vice versa (Gil et al., 2008; Li et al., 2002; Ma et al., 2022). In our experiment, simulations of low emitter discharge amounts (E1 and E2) were not as accurate as those of larger emitter discharge values (**Supplementary Table S2**), and a large emitter discharge was better adapted for fruit trees. This is because the horizontal transport of soil moisture relies mainly on substrate suction and its gravitational potential exceeds the substrate potential when the flow rate is too low. Thus, the rate of soil moisture transport in the vertical direction in this state is much greater than that in the horizontal transport. In addition, the large emitter discharge E4 saturates the surface soil with water after a period of water infiltration, and the rest of the water is transported to the deeper soil by gravity. Gravity promotes root water orientation in the vertical direction, helping roots search for water in the vertical direction, and inhibits root water orientation in the horizontal direction (Gao et al., 2024). As deep-rooted plants, the root system distribution range of fruit trees is relatively large and with a ‘narrow and deep’ wet zone, where there is a risk of deep seepage. The edge of this zone is likely to experience difficulties in absorbing root absorption, and the formation of a large emitter flow in the wet zone can occur in both the horizontal and vertical direction of the root system to absorb the water demand. This can effectively shorten the duration of irrigation. Emitter discharge and irrigation volume are prerequisites for promoting water use efficiency in fragrant pear by altering the inter-root soil moisture status and promoting water availability in the main root zone. Therefore, the establishment of irrigation regimes should be based on the spatial coordination of soil moisture and root distribution.

### 4.2 Effect of different irrigation treatments on soil salinity

In saline and arid regions, the principle of developing irrigation regimes must not only satisfy crop root zone water supply, but should also ensure that salinity levels within the root zone environment are controlled and that soil salinity stress is reduced to increase the production potential of fruit trees (Mair et al., 2023; Tan et al., 2023). Previous studies have shown that increasing water flux can promote salt leaching from the plant root zone and effectively reduce the stressful effects of salinity on the root system (Hou et al., 2022). In this study, soil salinity varied in a ‘sawtooth’ downward trend throughout the reproductive period, and the depth of the affected soil increased with the irrigation amount. This may be attributed to our use of a 15d high-frequency drip irrigation scheme, which to some extent offset the salt return effect caused by soil evaporation. The salt in the soil was gradually washed out of the root zone, resulting in an overall decrease in salt content. Furthermore, surface soil salinity fluctuations were the most variable, which may be due to the complex external environmental factors (e.g., temperature and meteorological factors) with the cumulative effects of soil evaporation and plant transpiration (Zhang et al., 2019). In addition, we observed a 21.30% reduction in salinity at the 100 cm depth under the W3 treatment compared to the W1 treatment for the same flow treatment (from the beginning to the end of the reproductive period). Note that we analyzed the salinity content of the 20–100 cm soil depth during the two-year fruit expansion period (when fragrant pear has the highest water demand and soil evaporation is the most intense) (**Figure 1**). The results showed that soil salinity exhibited a decreasing and subsequent increasing trend in the same irrigation period, forming a low-salt distribution domain in the range of 40–80 cm. Furthermore, the salt leaching range gradually moved to the deeper soil as the amount of irrigation increased (**Supplementary Figures S1J–1L**). Previous research has reported that the low-salinity distribution domain not only enhances water and nutrient uptake by the plant’s major absorptive roots (Zhang S. et al., 2021; Zhao et al., 2024), but that this region can also act as a salt barrier zone to reduce the detrimental upward migration of salts due to evaporation.

Numerous studies confirmed salinity as a limiting factor for the improvement of soil quality in arid areas. On the one hand, saline conditions affect the soil pore structure, leading to increased mechanical resistance to soil sloughing, which in turn affects root growth (Zhao et al., 2024). On the other hand, high soil salinity adversely affects nutrient content and microbial biomass, among other factors (AbuQamar et al., 2024). In this study, irrigation volume was negatively correlated with soil salinity levels, namely, increasing the irrigation amount promoted deep soil percolation while lowering the salinity threshold in the main root zone of fruit trees.

### 4.3 Irrigation strategy optimization and evaluation

**Figure 8:**
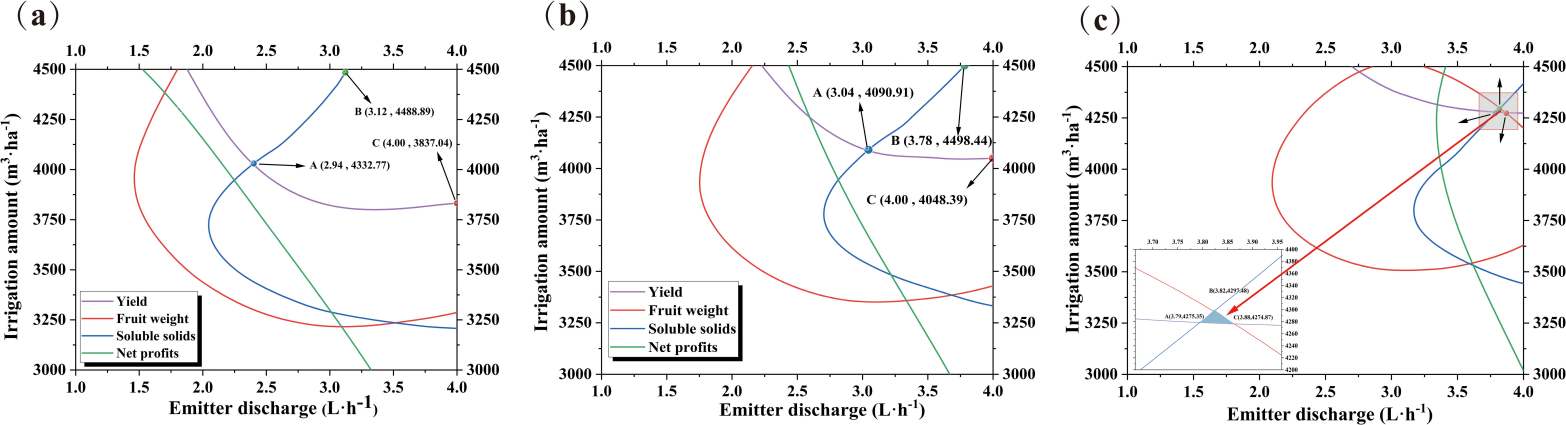
The relationship of the relative value of each evaluation index with the irrigation amounts and emitter discharge. **(A)**: The acceptable area is the relative value ≥ 70%; **(B)**: The acceptable area is the relative value ≥ 80%; **(C)**: The acceptable area is the relative value ≥ 90%.

Fruit yield, quality, and profitability are directly related to the economic income of farmers. Previous studies have confirmed that irrigation strategies have a significant effect on yield and quality (Chen et al., 2023; Lin et al., 2004). On the one hand, water deficit reduces the sap flow from the phloem to the xylem of the plant and the translocation of photosynthesis products to the fruit (i.e., the flow of water from the xylem to the fruit is reduced), which increases the concentration of solutes in the phloem. This can in turn enhance the fruit sugar content (Rodríguez-Celma et al., 2016). On the other hand, increasing irrigation volume can promote root growth and nutrient uptake to accelerate the photosynthetic rate, promote organic matter synthesis, and increase fruit yield (Wang et al., 2024; Zou et al., 2020). However, the continuous increase in irrigation volume not only increases economic expenditure and reduces the efficiency of water utilization, but also decreases fruit-reducing sugars and increases organic acids, which is detrimental to sugar accumulation and reduces fruit quality to some extent (Shu et al., 2020). Determining the optimal irrigation strategy is essential for achieving higher yields and better fruit quality, and a single indicator does not allow for a comprehensive evaluation of the overall economic performance of saline fruit trees. Therefore, it is necessary to establish a multi-objective decision-making model to maintain the water–salt environment of the fruit tree root system within a reasonable threshold range and to balance the relationship between yield and quality. This facilitates the optimization of the irrigation strategy to maximize the comprehensive benefits of planting. Scholars have solved for extreme values of the target by building multiple regression equations to accurately quantify and derive the optimal value of a target and the acceptable region for each indicator (Li et al., 2020; Wang et al., 2020). Due to the varying degrees of impact of experimental treatment on various indicators and differences in the unit dimensions of each evaluation indicator, it is not possible to use a single indicator as the evaluation standard. Therefore, in order to conduct a comprehensive evaluation, this article linearly normalizes the data of the above indicators and scales the data of each indicator according to the interval (0,1) (**Figure 7**). This article defines the regions where the maximum values of each evaluation index are ≥ 90%, ≥ 80%, and ≥ 70% as acceptable regions, that is, the boundaries of these three acceptable regions correspond to the contour lines of 0.90, 0.80, and 0.70 in (**Figure 8**), respectively. Based on spatial analysis, the contour lines of the above indicators are projected onto a plane to obtain a comprehensive evaluation analysis plan (**Figure 8**). In our study, the 90% overlap region was selected as the optimal irrigation and emitter discharge treatment interval as the envelope of the curves for each of the main objectives (i.e., fruit yield, fruit weight, soluble solids, and net profits) in the ≥80% and ≥70% acceptable regions was too large, resulting in deviations from the extremes. In contrast, the overlapping region for the ≥90% relative values accommodated the optimal values of each index. In addition, comprehensive modeling methods can effectively evaluate the spatial and temporal impacts of irrigation heterogeneity on field crop yield and soil moisture management under traditional and precision irrigation management strategies (González Perea et al., 2018, 2017). From an ecological perspective, compared with soil moisture management based schemes, plant physiological irrigation schemes can significantly reduce irrigation water use, increase economic profits, and improve irrigation water productivity without sacrificing crop yield (Zhang J. et al., 2021). Meanwhile, precise irrigation strategies can offset climate carbon feedback, increase carbon sequestration, and suppress soil respiration by maximizing environmental synergies or reducing thermal carbon emissions (Li P. et al., 2024). The optimal irrigation decision not only provides water resource utilization efficiency and crop yield quality, but also contributes to promoting sustainable agricultural development and dynamic adjustment and feedback.

Despite the great progress made by our study, it has some limitations. For example, we focused on the main root zone water and salt environments of a typical forest fruit in arid zones. However, as the root growth of fruit trees is a dynamic process, the root spatial configuration will be affected by the root zone soil environment, which needs to be targeted for the dynamic monitoring of the root system. Moreover, water and nutrient uptake by the root system is affected by soil solute potential, which needs to be investigated to determine the influence mechanism of soil solute potential on the root water uptake process. In addition, we investigated root zone soils with a focus on the water–salt environment, but the inter-feedback between the physicochemical properties of root zone soils, nutrient supply areas, microhabitats, and the root system also needs to be considered. Finally, future work should focus on the mechanisms driving the influence of root zone soil environmental factors on the root system and above-ground plant components.

## 5 Conclusion

This study is based on a 2-year surface drip irrigation experiment, using HYDRUS-2D to simulate the dynamic changes of soil water and salt in the root zone of saline alkali fruit trees. At the same time, spatial analysis was used to optimize irrigation strategies under multi-objective conditions, and the simulation results were verified from the perspective of production practice. The results showed that HYDRUS-2D can effectively simulate SWC and SSC in saline alkali root zone soil. The irrigation amount is positively correlated with soil infiltration depth and negatively correlated with SSC. Moderately increasing the irrigation amount can effectively leach salt from soil depths of 80-100 cm and maintain the water and salt environment in the main root zone. In addition, increasing the emitter discharge under the same irrigation amount can expand the horizontal moist range and form a “wide deep” moist area, improving the matching degree with the effective root zone. Furthermore, the multi-objective optimization method (i.e., fruit yield; fruit weight; soluble solids; net profits) has optimized the recommended irrigation range for early fruiting stage pears to be 4274-4297 m^3^ ha^-1^, and the drip head flow range to be 3.79-3.88 L h^-1^. These findings not only supplement and validate the numerical simulation results, but also broaden the comprehensive evaluation perspective of decision-makers. This study will provide a theoretical basis for us to develop irrigation systems for forests and fruits in saline alkali arid areas, improve fruit tree productivity, and promote agricultural production practices.

## Data Availability

The raw data supporting the conclusions of this article will be made available by the authors, without undue reservation.
