# Supplementary material for: Simulating water and salt changes in the root zone of salt–alkali fragrant pear and the selection of the optimal surface drip irrigation mode
Source: Front Plant Sci. 2024 Dec 11;15:1455188. doi: 10.3389/fpls.2024.1455188 (PMC11668977; doi:10.3389/fpls.2024.1455188)
Supplement: Supplementary file 1 [file DataSheet1.zip › 04 Supplementary material/01Supplementary figures.docx]

**Supplementary Figures Captions**

**Fig. S1.** Comparison of simulated and measured soil salinity values.





**Fig. S1.** Changes in SSC at depth for different treatments.
