# Supplementary material for: Simulating water and salt changes in the root zone of salt–alkali fragrant pear and the selection of the optimal surface drip irrigation mode
Source: Front Plant Sci. 2024 Dec 11;15:1455188. doi: 10.3389/fpls.2024.1455188 (PMC11668977; doi:10.3389/fpls.2024.1455188)
Supplement: Supplementary file 1 [file DataSheet1.zip › 04 Supplementary material/02 Supplementary tables.docx]

**Supplementary tables Captions**

**Table.S1** Water stress function parameters

**Table S2** The RMSE and NSE for SWC and SSC under different irrigation amount and emitter discharge strategies in 2021 (calibration) and 2022 (validation)

**Table. S3** Effects of different treatments of irrigation amounts and emitter discharges on economic benefits from 2021 and 2022

**Table.S1** Water stress function parameters

| Parameter | P_0_ (mm) | P_0_*_pt_* (mm) | P_2H_ (mm) | P_2L_ (mm) | P_3_ (mm) | r_2H_ (cm·d^-1^) | r_2L_ (cm·d^-1^) |
| --- | --- | --- | --- | --- | --- | --- | --- |
| Value | -100 | -250 | -5000 | -8000 | -80000 | 0.5 | 0.1 |

**Note:** The above parameters are referenced from the Wesseling (1991) database. Where P_0_ is the extracted water pressure head; P_0_*_pt_* is the maximum rate of extracted water pressure head; P_2H_ and P_2L_ are the limiting pressure heads; P_3_ is the apoplastic point pressure head; and r_2H_ and r_2L_ are the potential transpiration rates.

**Table S2** The RMSE and NSE for SWC and SSC under different irrigation amount and emitter discharge strategies in 2021 (calibration) and 2022 (validation)

| Year |  | 2021(Calibration) | | | | | | | | | | | | |
| --- | --- | --- | --- | --- | --- | --- | --- | --- | --- | --- | --- | --- | --- | --- |
| Treatment |  | W1 | | | | W2 | | | | W3 | | | | |
|  |  | E1 | E2 | E3 | E4 | E1 | E2 | E3 | E4 | E1 | E2 | E3 | E4 |  |
| SWC | RMSE (cm^3^·cm^-3^) | 0.09 | 0.08 | 0.16 | 0.08 | 0.04 | 0.09 | 0.09 | 0.07 | 0.06 | 0.03 | 0.10 | 0.05 |  |
|  | NSE | 0.91 | 0.83 | 0.83 | 0.92 | 0.96 | 0.83 | 0.76 | 0.92 | 0.79 | 0.85 | 0.93 | 0.87 |  |
| SSC | RMSE (g·kg^-1^) | 0.96 | 0.44 | 0.48 | 0.35 | 0.27 | 0.22 | 0.87 | 0.25 | 0.15 | 0.74 | 0.84 | 0.22 |  |
|  | NSE | 0.85 | 0.91 | 0.68 | 0.96 | 0.96 | 0.92 | 0.95 | 0.85 | 0.93 | 0.86 | 0.92 | 0.89 |  |
| Year |  | 2022(Validation) | | | | | | | | | | | | |
| Treatment |  | W1 | | | | W2 | | | | W3 | | | | |
|  |  | E1 | E2 | E3 | E4 | E1 | E2 | E3 | E4 | E1 | E2 | E3 | E4 |  |
| SWC | RMSE (cm^3^·cm^-3^) | 0.15 | 0.11 | 0.08 | 0.05 | 0.12 | 0.13 | 0.15 | 0.02 | 0.15 | 0.09 | 0.11 | 0.04 |  |
|  | NSE | 0.86 | 0.92 | 0.95 | 0.95 | 0.89 | 0.90 | 0.69 | 0.98 | 0.82 | 0.95 | 0.91 | 0.84 |  |
| SSC | RMSE (g·kg^-1^) | 0.85 | 1.54 | 0.38 | 0.99 | 0.39 | 0.88 | 1.04 | 0.38 | 0.75 | 0.67 | 0.73 | 0.98 |  |
|  | NSE | 0.94 | 0.70 | 0.72 | 0.93 | 0.93 | 0.85 | 0.88 | 0.86 | 0.77 | 0.83 | 0.93 | 0.89 |  |

**Table S3** Effects of different treatments of irrigation amounts and emitter discharges on economic benefits from 2021 and 2022

| Year | Treatment | Gp (RMB·ha^-1^) | Wc (RMB·ha^-1^) | Fc (RMB·ha^-1^) | L (RMB·ha^-1^) | Np (RMB·ha^-1^) |
| --- | --- | --- | --- | --- | --- | --- |
| 2021 | W1E1 | 70648.98 | 2100 | 2740 | 27000 | 38808.98 ± 382.48 Cd |
|  | W1E2 | 72689.57 | 2100 | 2740 | 27000 | 40849.57 ± 897.41 Cc |
|  | W1E3 | 73115.46 | 2100 | 2740 | 27000 | 41275.46 ± 526.03 Cb |
|  | W1E4 | 74412.31 | 2100 | 2740 | 27000 | 42572.31 ± 327.22 Ca |
|  | W2E1 | 76892.55 | 2625 | 2740 | 29000 | 42527.55 ± 653.96 Bd |
|  | W2E2 | 77420.63 | 2625 | 2740 | 29000 | 43055.63 ± 623.86 Bc |
|  | W2E3 | 81728.52 | 2625 | 2740 | 29000 | 47363.52 ± 93.07 Bb |
|  | W2E4 | 86545.82 | 2625 | 2740 | 29000 | 52180.82 ± 808.47 Ba |
|  | W3E1 | 82785.81 | 3150 | 2740 | 31000 | 45895.81 ± 388.77 Ad |
|  | W3E2 | 86613.53 | 3150 | 2740 | 31000 | 49723.53 ± 1095.50 Ac |
|  | W3E3 | 88231.91 | 3150 | 2740 | 31000 | 51341.91 ± 390.21 Ab |
|  | W3E4 | 93763.92 | 3150 | 2740 | 31000 | 56873.92 ± 126.85 Aa |
| 2022 | W1E1 | 80223.00 | 2100 | 2400 | 27000 | 48723.00 ± 1941.32 Cd |
|  | W1E2 | 86770.28 | 2100 | 2400 | 27000 | 55270.28 ± 963.38 Cc |
|  | W1E3 | 88047.94 | 2100 | 2400 | 27000 | 56547.94 ± 779.85 Cb |
|  | W1E4 | 93156.36 | 2100 | 2400 | 27000 | 61656.36 ± 451.65 Ca |
|  | W2E1 | 89521.25 | 2625 | 2400 | 29000 | 55496.25 ± 1516.36 Bd |
|  | W2E2 | 90262.80 | 2625 | 2400 | 29000 | 56237.80 ± 1369.02 Bc |
|  | W2E3 | 100665.00 | 2625 | 2400 | 29000 | 66640.02 ± 933.70 Bb |
|  | W2E4 | 91584.73 | 2625 | 2400 | 29000 | 57559.73 ± 1556.09 Ba |
|  | W3E1 | 94810.90 | 3150 | 2400 | 31000 | 58260.90 ± 1423.60 Ad |
|  | W3E2 | 96185.20 | 3150 | 2400 | 31000 | 59635.20 ± 1410.50 Ac |
|  | W3E3 | 101190.00 | 3150 | 2400 | 31000 | 64640.02 ± 885.66 Ab |
|  | W3E4 | 105458.50 | 3150 | 2400 | 31000 | 68908.48 ± 634.40 Aa |

**Note:** where is the gross *G_p_* is the gross profit; *W_c_* is the water and electricity cost; *F_c_* is the fertilizer cost; and *L* denotes other costs such as field management costs, pesticide costs, weed control costs and labor costs. Capital letters indicate differences between different irrigation volumes and lowercase letters indicate differences between different emitter discharge.
